# Quantitative MRI distinguishes different leukodystrophies and correlates with clinical measures

**DOI:** 10.1007/s00330-024-11089-5

**Published:** 2024-09-25

**Authors:** Menno D. Stellingwerff, Murtadha L. Al-Saady, Kwok-Shing Chan, Adam Dvorak, José P. Marques, Shannon Kolind, Daphne H. Schoenmakers, Romy van Voorst, Stefan D. Roosendaal, Frederik Barkhof, Nicole I. Wolf, Johannes Berkhof, Petra J. W. Pouwels, Marjo S. van der Knaap

**Affiliations:** 1 Amsterdam Leukodystrophy Center, Department of Child Neurology, Emma Children’s Hospital, Amsterdam University Medical Centers, and Amsterdam Neuroscience, Cellular & Molecular Mechanisms, Vrije Universiteit, Amsterdam, The Netherlands; 2 Donders Institute for Brain, Cognition and Behaviour, Radboud University, Nijmegen, The Netherlands; 3 Athinoula A. Martinos Center for Biomedical Imaging, Massachusetts General Hospital, Charlestown, MA USA; 4 Department of Radiology, Harvard Medical School, Boston, MA USA; 5 Department of Physics and Astronomy, University of British Columbia, Vancouver, BC Canada; 6 Medicine for Society, Platform at Amsterdam UMC, University of Amsterdam, Amsterdam, The Netherlands; 7 Department of Radiology and Nuclear Medicine, Amsterdam University Medical Centers and Amsterdam Neuroscience, Amsterdam, The Netherlands; 8 Institutes of Neurology and Healthcare Engineering, University College London, London, UK; 9 Department of Epidemiology and Data Science, Amsterdam UMC Location Vrije Universiteit, Amsterdam, The Netherlands; 10 Department of Integrative Neurophysiology, Center for Neurogenomics and Cognitive Research, Vrije Universiteit, Amsterdam, The Netherlands

**Keywords:** Magnetic resonance imaging, Brain, Pediatrics and adults, Vanishing white matter, Metachromatic leukodystrophy

## Abstract

**Objectives:**

The leukodystrophy “vanishing white matter” (VWM) and “metachromatic leukodystrophy” (MLD) affect the brain's white matter, but have very different underlying pathology. We aim to determine whether quantitative MRI reflects known neuropathological differences and correlates with clinical scores in these leukodystrophies.

**Methods:**

VWM and MLD patients and controls were prospectively included between 2020 and 2023. Clinical scores were recorded. MRI at 3 T included multi-compartment relaxometry diffusion-informed myelin water imaging (MCR-DIMWI) and multi-echo T2-relaxation imaging with compressed sensing (METRICS) to determine myelin water fractions (MWF). Multi-shell diffusion-weighted data were used for diffusion tensor imaging measures and neurite orientation dispersion and density imaging (NODDI) analysis, which estimates neurite density index, orientation dispersion index, and free water fraction. As quantitative MRI measures are age-dependent, ratios between actual and age-expected MRI measures were calculated. We performed the multilevel analysis with subsequent post-hoc and correlation tests to assess differences between groups and clinico-radiological correlations.

**Results:**

Sixteen control (age range: 2.3–61.3 years, 8 male), 37 VWM (2.4–56.5 years, 20 male), and 14 MLD (2.2–41.7 years, 6 male) subjects were included. Neurite density index and MWF were lower in patients than in controls (*p* < 0.001). Free water fraction was highest in VWM (*p* = 0.01), but similar to controls in MLD (*p* = 0.99). Changes in diffusion tensor imaging measures relative to controls were generally more pronounced in VWM than in MLD. In both patient groups, MCR-DIMWI MWF correlated strongest with clinical measures.

**Conclusion:**

Quantitative MRI correlates to clinical measures and yields differential profiles in VWM and MLD, in line with differences in neuropathology.

**Key Points:**

***Question*** *Can quantitative MRI reflect known neuropathological differences and correlate with clinical scores for these leukodystrophies*?

***Finding*** *Quantitative MRI measures, e.g., MWF, neurite density index, and free water fraction differ between leukodystrophies and controls, in correspondence to known histological differences*.

***Clinical relevance*** *MRI techniques producing quantitative, biologically-specific, measures regarding the health of myelin and axons deliver more comprehensive information regarding pathological changes in leukodystrophies than current approaches, and are thus viable tools for monitoring patients and providing clinical trial outcome measures*.

## Introduction

Leukodystrophies constitute a large group of heterogeneous genetic diseases, which mainly affect the white matter of the CNS [1, 2]. MRI has had an enormous impact on the leukodystrophy field by greatly facilitating the diagnosis, mostly by pattern recognition using conventional T2-weighted, T1-weighted, and fluid-attenuated inversion recovery (FLAIR) images [3]. Conventional MRI is, however, suboptimal for monitoring changes in tissue structure with disease progression and in response to treatment. Quantitative MRI techniques attempt to provide imaging metrics with high specificity to underlying tissue changes. Recently, we developed and implemented a scanning protocol for measuring quantitative tissue parameters in leukodystrophies, including myelin water fraction (MWF), neurite density index (NDI), and diffusion tensor imaging (DTI) measures, which have each shown promise at better disentangling pathological processes [4]. As a first step, we showed that these methods are clinically feasible and can distinguish between control subjects and patients with leukodystrophy [5].

Two of the more prevalent leukodystrophies are vanishing white matter (VWM, OMIM 603896) and metachromatic leukodystrophy (MLD, OMIM 250100) [6, 7]. VWM is caused by biallelic pathogenic variants in any of the five genes *EIF2B1–EIF2B5* encoding eukaryotic initiation factor 2B (eIF2B) [8]. MLD is caused by biallelic pathogenic variants in *ARSA* encoding the lysosomal enzyme arylsulfatase A [9]. Although the cerebral white matter is extensively affected in both diseases, the pathophysiological mechanism and neuropathological findings differ considerably. VWM is characterized by a defect in the integrated stress response, resulting in dysfunction of astrocytes and oligodendrocytes [10, 11]. This causes progressive degeneration of the cerebral white matter with loss of all components and very little gliosis, leading to rarefaction and cystic decay [12]. MLD is a lysosomal storage disease characterized by the accumulation of sulfatide due to the deficiency of arylsulfatase A [9]. Sulfatides are membrane lipids with the highest concentration in myelin; accumulation primarily causes demyelination with relative preservation of axons. Lysosomal accumulation of sulfatides occurs mostly in astrocytes and macrophages. There is a dense gliosis [2].

In this study, we aimed to determine whether the known differences in neuropathology between VWM and MLD are reflected in differential changes in quantitative MRI measures and whether such measures correlate with clinical severity scores, which would make them suitable for monitoring patients and for serving as outcome measures in trials.

## Materials and methods

### Participants and clinical measures

This prospective study was approved by the local ethics committee and signed informed consent was obtained from subjects and/or caregivers. Patients were consecutively included between 2020 and 2023 and had a genetically confirmed diagnosis of VWM or MLD. Patients with a second genetic disease [6, 7] were excluded. Age and disease duration were recorded. Three measures were assessed to capture the patients’ clinical picture. The Health Utilities Index (HUI) estimates the level of functioning in daily life [13]. The Vineland-3 adaptive behavior composite (ABC) score quantifies performance in the domains of communication, daily life skills, and socialization [14]. The GMFC-MLD is a gross motor function score, developed for MLD, but also applicable in other leukodystrophies [15]. The scans of patients have not been previously reported.

Most scans of control subjects (15 out of 16) have been previously reported [5]. This prior article dealt with the development of the MRI acquisition protocol used in the current manuscript. Control subjects were control participants or patients scanned because of minor neurological complaints, such as headache/migraine, long-standing vomiting, or syncope. Control subjects with white matter signal abnormalities on conventional MRI were excluded.

### MRI acquisition protocol

MRI scans were obtained on a 3-T scanner (Philips Ingenia). The acquisition protocol has been described in detail [5] and consisted of 3D T1-weighted and 3D FLAIR images at 0.9–1.0 mm isotropic resolution, and three quantitative sequences at 2.5 mm isotropic resolution: (I) 2D multi-shell diffusion-weighted imaging [DWI] with *b*-values 0 s/mm^2^, 1000 s/mm^2^, 2000 s/mm^2^, (II) 3D multi-gradient echo with variable flip angles [MGRE-VFA] with seven flip angles [5, 10, 20, 30, 40, 50, and 70] and 12 echo times [2.15–35.70 ms, step size 3.05 ms], and (III) 3D multi-echo T2 relaxation imaging with compressed sensing [METRICS], 56 echo times [7–392 ms, step size 7 ms] [16, 17]. DWI data were used to determine DTI measures based on a single b1000-shell, and neurite orientation dispersion and density imaging (NODDI) measures based on all shells. NODDI estimates the NDI, orientation dispersion index (ODI), and free (isotropic) water fraction (FISO) [18]. MGRE-VFA data were used for single-compartment T1 and T2* maps and, in combination with multi-shell DWI data, for multi-compartment relaxometry diffusion-informed myelin water imaging (MCR-DIMWI) [16]. MCR-DIMWI and METRICS both estimate the MWF. METRICS also gives the geometrical mean of the intra- and extra-axonal T2 (IET2) as output.

### MRI post-processing

MRI post-processing was performed as reported previously [5]. We applied an automatic anatomy-based pipeline independent of signal abnormalities or arbitrary cut-off values. In short, 3D T1 weighted images were segmented into anatomical white matter, cortical gray matter, deep gray matter, and cerebrospinal fluid using Synthseg [19]. Manual correction was performed when needed. DWI was co-registered to IIT space (atlas space of Illinois Institute of Technology) using dtitk [20]. Synthseg segmentations were combined with the MNI brain atlas (Montreal Neurological Institute) to define white matter within the cerebral lobes. Median values were obtained from six white matter regions of interest (ROIs): total cerebral, left frontal, left parietal, left occipital, left temporal, and left cerebellar white matter. In addition, weighted means were obtained from two probabilistic tracts defined in the IIT atlas: the left corticospinal tract and the tracts passing through the corpus callosum [21].

To explore the influence of pathology in MLD and VWM on T2 distribution curves, we randomly selected three control, three VWM, and three MLD subjects. We delineated small ROIs with (a) normal-appearing white matter (FLAIR-hypointense and T1-hyperintense compared to cortex signal intensity), (b) abnormal non-rarefied white matter (FLAIR-hyperintense and T1-hypointense compared to cortex signal intensity), and (c) rarefied to cystic white matter (lower FLAIR signal intensity than normal-appearing white matter). Signal decay curves of the voxels were averaged to enable one METRICS fit per ROI, which subsequently yielded one T2 distribution per ROI per subject.

### Statistical analysis

Statistical analyses were performed with RStudio (RStudio Team, version 1.1.463). The normality of data was assessed with the Shapiro–Wilk test. Normally distributed data are displayed as mean ± standard deviation, and non-normally distributed data as median (range).

Age-related changes occur for MRI measures and roughly follow asymptotic increase or decline from infancy to (early) adulthood [22, 23]. To correct for age-related changes and facilitate comparison between groups, a parameter-specific and ROI-specific asymptotic fit (α − (α − β) × exp(τ × age)) was made through control values. In this fit, α represents the plateau value at adult age, β the value at birth, and τ the steepness of the increase or decrease with age. Using the derived parameters α, β, and τ, an expected value could be determined for each subject based on age at MRI. For each measure in each subject, we calculated the ratio between observed and expected values for age (O/E).

As each subject had data points from eight ROIs, multilevel analyses were performed. Linear mixed models, with O/E as the dependent variable, and the subject as the clustering variable, were used to determine whether an MRI measure differed between groups when including all ROIs. If group differences were found, we applied Tukey’s post-hoc tests to determine which groups differed from each other. We analyzed ROIs separately with ANOVAs to determine whether groups and which groups differed within a specific ROI. Holm multiple testing correction was used when appropriate.

Correlations between quantitative MRI measures and clinical scores were analyzed using Pearson correlation for HUI and Vineland-3 ABC scores, and Spearman correlation for GMFC-MLD. All statistical tests were performed two-sided and a *p*-value of < 0.05 was considered statistically significant.

## Results

### Visual results of MRI images and quantitative maps

**Fig. 1:**
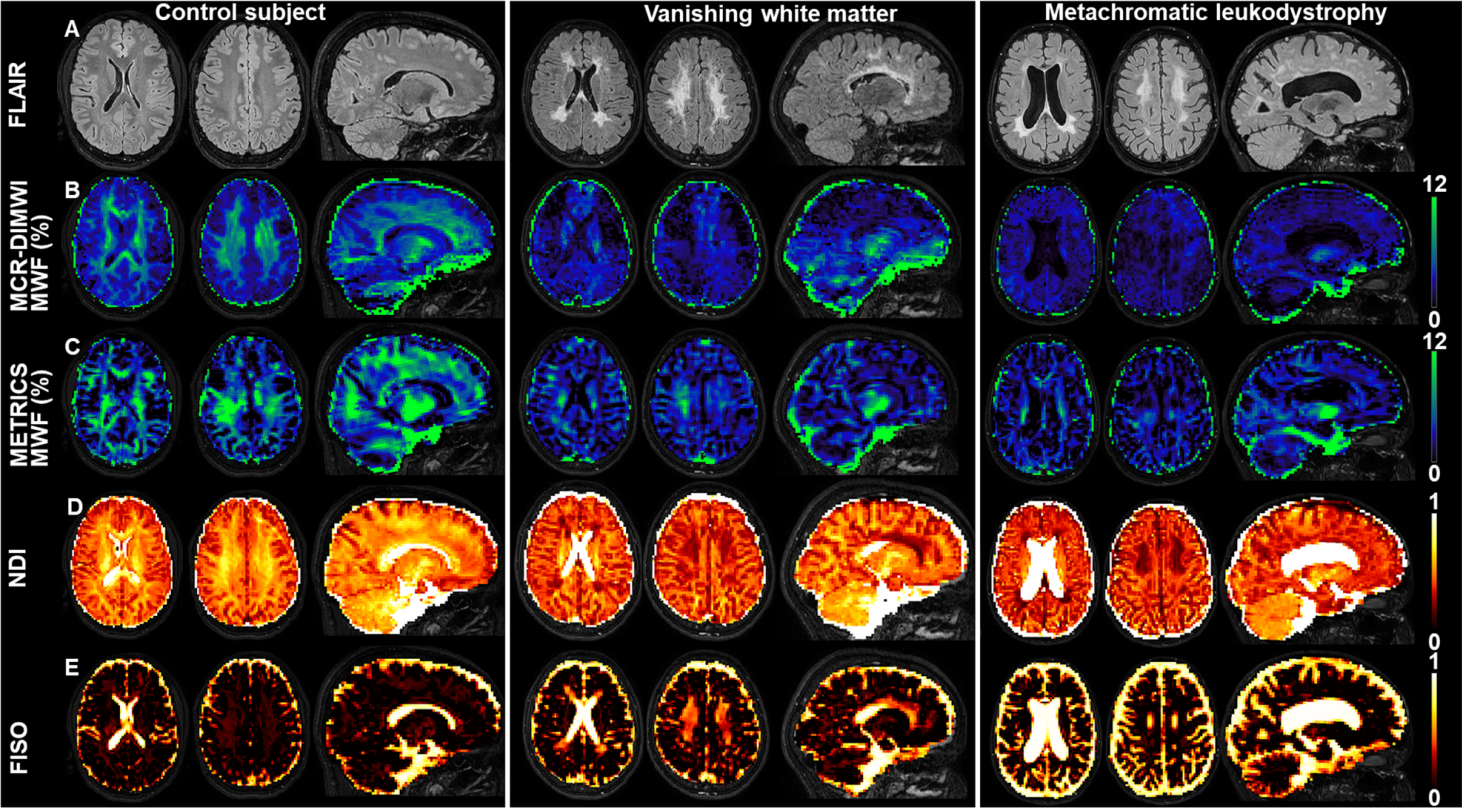
FLAIR images and quantitative MRI maps. The left columns show images of a control subject (13 years), the middle columns of a VWM patient (12 years), and the right columns of an MLD patient (15 years). Areas with FLAIR hyperintense signal intensities are seen in the patient with VWM and in the patient with MLD (**A**), indicating abnormal white matter. In the patient with VWM, small areas of rarefied white matter (lower FLAIR signal intensity than normal-appearing white matter) are seen in the frontal white matter. The MCR-DIMWI- and METRICS-derived MWF (respectively, **B**, **C**) are lower in the two patients than in the control subject, especially in affected white matter areas. Furthermore, NDI (**D**) is lower in the two patients than in the control subject, although the cerebellar white matter looks normal in the VWM subject. Despite the limited clear rarefaction and the relatively similar appearance on FLAIR of the VWM and MLD patient, FISO (**E**) is higher in the patient with VWM, than in the patient with MLD and the control subject. Additional maps (FA, MD, RD, T2*, T1, and IET2) are available online in Fig. S1. FLAIR, fluid-attenuated inversion recovery; VWM, vanishing white matter; MLD, metachromatic leukodystrophy; MWF, myelin-water fraction; MCR-DIMWI, multi-compartment relaxometry diffusion-informed myelin water imaging; METRICS, multi-echo T2 relaxation imaging with compressed sensing; FISO, free (isotropic) water fraction; NDI, neurite density index

MRI scans were obtained from 16 control subjects, 37 VWM patients, and 14 MLD patients (Table 1). Due to rarefied/cystic areas adjacent to the ventricles, automatic segmentations in VWM sometimes had to be manually adjusted to ensure correct delineation of the lateral ventricles. Figure 1 shows FLAIR images and quantitative MRI maps in a control, VWM, and MLD subject. FLAIR hyperintense white matter was seen in both VWM and MLD, whereas rarefaction to cystic white matter was mainly seen in VWM (Fig. 1A). The MCR-DIMWI- and METRICS-derived MWF (Fig. 1B, C) was lower in VWM and MLD than in controls and was spatially correlated to affected white matter on FLAIR. NDI (Fig. 1D) was lower in patients than in controls. FISO (Fig. 1E) was often high in white matter in VWM and normal in MLD compared to controls. Additional quantitative maps are shown in Fig. S1.

**Table 1:** Demographic and clinical characteristics

Group	Controls, (*n* = 16)	VWM, (*n* = 37)	MLD, (*n* = 14)
*N* (%) males	8 (50%)	20 (54%)	6 (43%)
Age MRI, median (range)	25.0 (2.3–61.3)	7.9 (2.4–56.5)	23.8 (2.2–41.7)
Age of onset, median (range)		4 (1–50)	12 (2–28)
Disease duration, median (range)		3.2 (0.2–25.4)	8.8 (0.2–20.5)
*N* treated with HSCT (%)	NA	NA	8 (57%)
GMFC-MLD (*n* per level (%))	NA	NA: 2 (5%) M0: 10 (27%) M1: 21 (57%) M2: 4 (11%)	NA: 1 (7%) M0: 2 (14%) M1: 9 (64%) M2: 2 (14%)
Vineland-3 ABC, median (range)	NA	77 (22–108)	56 (20–103)
HUI, median (range)	NA	0.63 (0.13–1.00)	0.21 (−0.23 to 0.86)

Patients with GMFC-MLD M3–M6 were not present in this cohort

*VWM* vanishing white matter, *MLD* metachromatic leukodystrophy, *n* number, *HSCT* hematopoietic stem cell transplantation, *GMFC-MLD* gross motor function classification score for MLD, *NA* not assessed/not applicable, *M0* walking without support with quality of performance normal for age, *M1* walking without support but with reduced quality of performance, i.e., instability when standing or walking, *M2* walking with support. Walking without support is not possible (fewer than five steps), *Vineland-3 ABC* Vineland-3 overall adaptive behavior composite score, *HUI* generic health utility index score

### Quantitative comparison

**Fig. 2:**
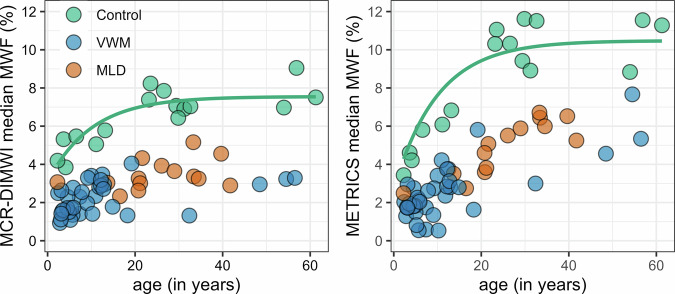
MWF in cerebral white matter vs age. Median MCR-DIMWI- and METRICS-derived MWF of control subjects (green), VWM patients (blue), and MLD patients (orange) in cerebral white matter vs age. A mono-exponential fit is displayed through the control values (green line). Additional MRI measures and ROIs are available online in Fig. S2. VWM, vanishing white matter; MLD, metachromatic leukodystrophy; MWF, myelin-water fraction; ROI, region-of-interest; MCR-DIMWI, multi-compartment relaxometry diffusion-informed myelin water imaging; METRICS, multi-echo T2 relaxation imaging with compressed sensing

Figure 2 shows a clear age effect in the MCR-DIMWI- and METRICS-derived MWF in cerebral white matter in controls (MCR-DIMWI *F*(1,14) = 20.8, *p* < 0.001; METRICS *F*(1,14) = 21.5, *p* < 0.001). For both techniques, MWF was lower in VWM and MLD than in controls. Quantitative values per ROI of other MRI measures are presented in Fig. S2 and Table S1. The O/E ratios are shown in Table S2.

**Fig. 3:**
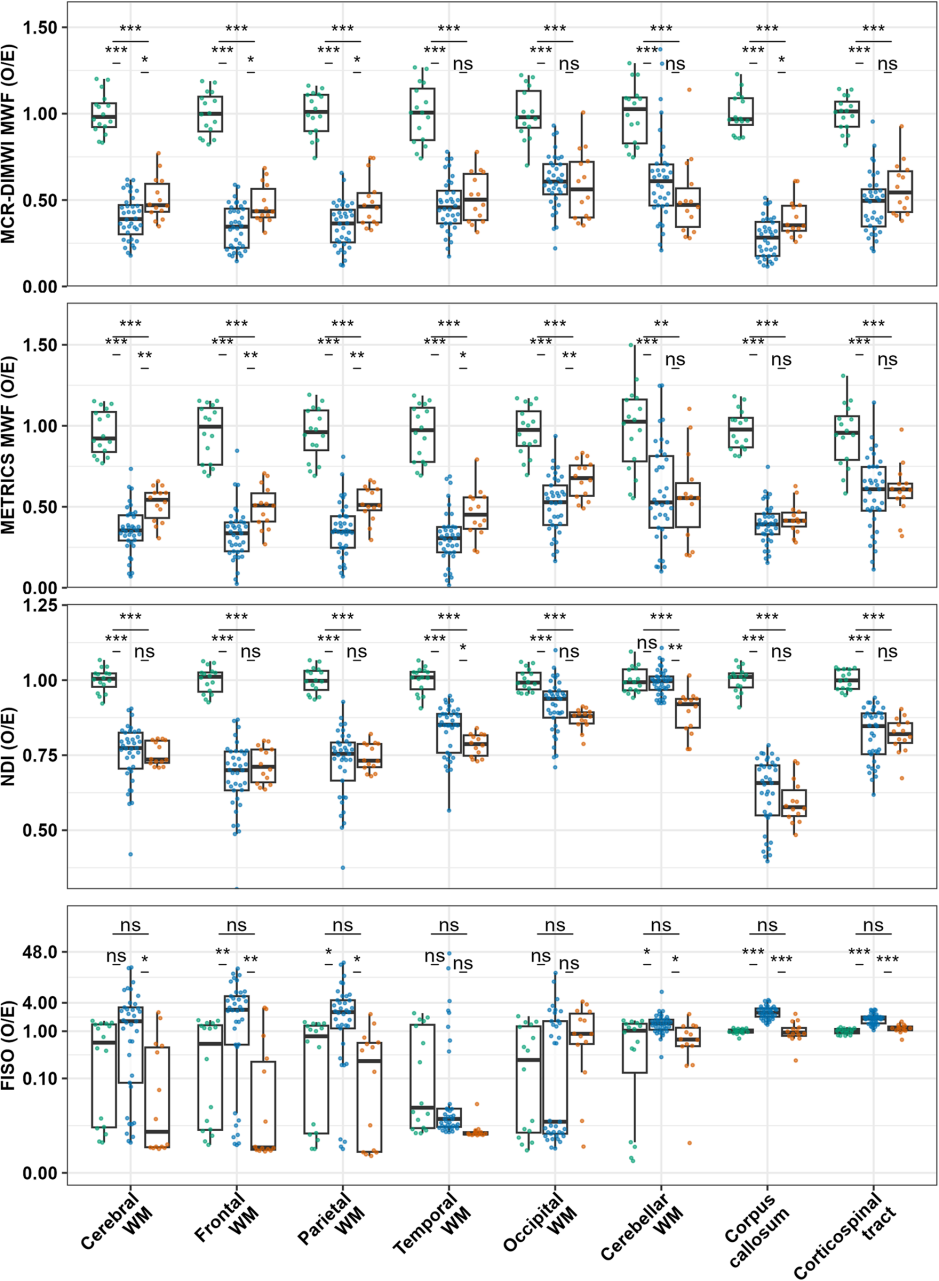
Differences of age-corrected quantitative MRI ratios. Boxplots of the ratio of the O/E value in controls (green), patients with VWM (blue), and patients with MLD (orange). ns, not significant, *p < 0.05, **p < 0.01, ***p < 0.001. The scale for FISO is logarithmic. The corpus callosum and corticospinal ROIs are tract-based ROIs. Additional MRI measures and ROIs are available online in Fig. S3. VWM, vanishing white matter; MLD, metachromatic leukodystrophy; ROI, region-of-interest; FISO, free (isotropic) water fraction

Multi-level testing across regions indicated group differences in MWF in both MCR-DIMWI and METRICS (both *p* < 0.001, Table 2): MWF was lower in VWM and MLD than controls (each *p* < 0.001), but did not differ between VWM and MLD for MCR-DIMWI (*p* = 0.28) and METRICS (*p* = 0.08). In all ROIs, MWF was lower in VWM and MLD than in controls; in most ROIs, MWF was lower in VWM than in MLD (Table 3 and Fig. 3).

**Table 2:** Group differences per MRI measure

Quantitative MRI measure	Group difference *F*-value, *p*-value	VWM vs control *t*-ratio, *p*-value	MLD vs control *t*-ratio, *p*-value	VWM vs MLD *t*-ratio, *p*-value
MCR-DIMWI MWF	109.4, < 0.001	−14.5, < 0.001	−10.6, < 0.001	−**1.5, 0.280**
T1	47.4, < 0.001	9.6, < 0.001	3.9, < 0.001	4.6, < 0.001
T2*	40.4, < 0.001	8.7, < 0.001	3.1, 0.008	4.7, < 0.001
METRICS MWF	70.7, < 0.001	−11.8, < 0.001	−7.8, < 0.001	−**2.2, 0.081**
IET2	84.5, < 0.001	12.9, < 0.001	6.4, < 0.001	4.9, < 0.001
MD	38.3, < 0.001	8.7, < 0.001	4.9, < 0.001	2.6, 0.028
RD	49.4, < 0.001	9.9, < 0.001	5.7, < 0.001	2.9, 0.015
AD	19.1, < 0.001	6.2, < 0.001	3.4, 0.004	**2.0, 0.129**
FA	123.6, < 0.001	−15.7, < 0.001	−9.5, < 0.001	−4.0, < 0.001
NDI	63.6, < 0.001	−10.5, < 0.001	−9.4, < 0.001	**1.0, 0.594**
ODI	27.1, < 0.001	7.2, < 0.001	2.8, 0.016	3.5, 0.002
FISO	4.9, 0.010	2.5, 0.035	**0.0, 0.999**	2.5, 0.041

Non-significance is marked using bold font

*MCR-DIMWI* multi-compartment relaxometry-diffusion informed myelin water imaging, *METRICS* multi-echo T2 relaxation imaging with compressed sensing, *MWF* myelin water fraction, *IET2* geometrical mean of the intra- and extra-axonal T2, *MD* mean diffusivity, *RD* radial diffusivity, *AD* axial diffusivity, *FA* fractional anisotropy, *NDI* neurite density index, *ODI* orientation dispersion index, *FISO* free water fraction, *VWM* vanishing white matter, *MLD* metachromatic leukodystrophy

**Table 3:** Group differences per MRI measure per region of interest

		MCR-DIMWI			METRICS		DTI				NODDI		
	Group comparison	MWF	T1	T2*	MWF	IET2	MD	RD	AD	FA	NDI	ODI	FISO
Cerebral WM	VWM vs control	−16.7, ***	12.8, ***	11.2, ***	−14.3, ***	16.6, ***	10.1, ***	11.7, ***	7.3, ***	−19.7, ***	−12.8, ***	10.6, ***	**3.0, n.s**.
	MLD vs control	−11.1, ***	8.9, ***	9.2, ***	−9.8, ***	10.3, ***	9.0, ***	10.2, ***	5.6, ***	−9.8, ***	−16.9, ***	3.7, *	**−** **1.1, n.s**.
	VWM vs MLD	−2.8, *	5.3, ***	5.9, ***	−4.3, **	5.7, ***	2.9, *	3.0, *	**2.2, n.s**.	−4.5, **	**0.0, n.s**.	3.3, *	3.3, *
Frontal WM	VWM vs control	−17.4, ***	13.3, ***	11.2, ***	−12.1, ***	20.6, ***	10.8, ***	11.8, ***	8.8, ***	−21.3, ***	−14.5, ***	7.3, ***	4.3, **
	MLD vs control	−12.1, ***	8.2, ***	6.7, ***	−8.1, ***	9.3, ***	7.3, ***	8.4, ***	5.0, **	−10.0, ***	−14.5, ***	**2.6, n.s**.	**−** **0.6, n.s**.
	VWM vs MLD	−3.5, *	6.5, ***	6.9, ***	−3.7, **	6.6, ***	3.7, **	3.6, **	3.5, *	−4.5, **	**−** **1.5, n.s**.	**2.5, n.s**.	4.4, **
Parietal WM	VWM vs control	−16.8, ***	12.6, ***	9.8, ***	−12.2, ***	14.5, ***	9.6, ***	10.9, ***	6.9, ***	−17.1, ***	−12.5, ***	9.8, ***	3.5, *
	MLD vs control	−10.1, ***	7.6, ***	8.3, ***	−8.6, ***	8.8, ***	8.6, ***	9.5, ***	5.5, **	−8.8, ***	−15.7, ***	3.6, *	−**1.0, n.s**.
	VWM vs MLD	−3.0, *	5.8, ***	5.7, ***	−4.1, **	6.0, ***	3.6, **	3.7, **	**2.8, n.s**.	−5.4, ***	**−** **0.9, n.s**.	4.0, **	3.7, *
Temporal WM	VWM vs control	−10.7, ***	11.2, ***	10.6, ***	−12.5, ***	15.6, ***	7.4, ***	9.8, ***	**2.3, n.s**.	−15.5, ***	−9.3, ***	11.4, ***	**1.1, n.s**.
	MLD vs control	−8.4, ***	7.8, ***	7.7, ***	−8.3, ***	11.3, ***	9.1, ***	10.9, ***	**2.8, n.s**.	−9.9, ***	−14.6, ***	4.5, **	**−** **2.8, n.s**.
	VWM vs MLD	**−** **1.0, n.s**.	4.0, **	3.8, **	−3.0, *	4.0, **	**1.0, n.s**.	**1.6, n.s**.	**0.1, n.s**.	−3.3, **	2.9, *	3.4, *	**1.6, n.s**.
Occipital WM	VWM vs control	−8.7, ***	7.2, ***	9.9, ***	−9.5, ***	7.9, ***	5.9, ***	7.3, ***	3.8, **	−7.5, ***	−5.1, ***	5.9, ***	**1.6, n.s**.
	MLD vs control	−6.3, ***	4.5, **	8.4, ***	−6.0, ***	9.9, ***	6.0, ***	6.0, ***	6.0, ***	−4.7, **	−9.9, ***	**2.1, n.s**.	**2.1, n.s**.
	VWM vs MLD	**0.4, n.s**.	2.7, *	2.3, *	−3.6, **	**1.0, n.s**.	**−** **1.1, n.s**.	**−** **0.4, n.s**.	**−** **2.4, n.s**.	**−** **0.7, n.s**.	**2.5, n.s**.	**2.1, n.s**.	**0.1, n.s**.
Cerebellar WM	VWM vs control	−6.1, ***	4.7, ***	3.7, **	−4.8, ***	5.3, ***	4.6, ***	6.2, ***	**1.9, n.s**.	−7.1, ***	**−** **0.6, n.s**.	6.7, ***	3.2, *
	MLD vs control	−6.5, ***	4.3, **	−0.3, **	−4.3, **	4.2, **	4.8, **	5.9, ***	**2.4, n.s**.	−6.8, ***	−4.9, ***	3.6, *	**−** **0.3, n.s**.
	VWM vs MLD	**1.5, n.s**.	**0.2, n.s**.	**−** **1.8, n.s**.	**0.2, n.s**.	**−** **1.1, n.s**.	**−** **1.9, n.s**.	**−** **1.8, n.s**.	**−** **1.3, n.s**.	**1.5, n.s**.	4.9, **	**1.1, n.s**.	3.5, *
Corpus callosum	VWM vs control	−20.7, ***	14.4, ***	12.3, ***	−15.7, ***	24.4, ***	13.4, ***	15.2, ***	8.2, ***	−25.6, ***	−17.0, ***	9.8, ***	11.7, ***
	MLD vs control	−14.4, ***	8.9, ***	6.7, ***	−13.7, ***	9.7, ***	9.2, ***	11.0, ***	3.8, *	−14.9, ***	−18.2, ***	5.1, **	**0.5, n.s**.
	VWM vs MLD	−3.2, *	5.4, ***	6.4, ***	**−** **1.0, n.s**.	4.7, ***	3.3, *	3.2, *	**2.8, n.s**.	−3.4, **	**1.1, n.s**.	**2.6, n.s**.	7.9, ***
Corticospinal tract	VWM vs control	−13.7, ***	11.4, ***	9.2, ***	−6.0, ***	16.7, ***	13.4, ***	13.3, ***	11.7, ***	−12.3, ***	−10.9, ***	3.3, *	10.2, ***
	MLD vs control	−8.8, ***	9.6, ***	7.9, ***	−5.3, ***	8.6, ***	8.7, ***	8.8, ***	6.7, ***	−7.6, ***	−10.3, ***	**−** **1.1, n.s**.	**2.3, n.s**.
	VWM vs MLD	**−** **1.8, n.s**.	6.5, ***	6.2, ***	**−** **0.4, n.s**.	5.6, ***	4.7, ***	4.9, ***	3.4, *	−4.9, ***	**0.0, n.s**.	3.9, **	7.3, ***

n.s., non-significance, and is marked using bold font. The *t*-ratio and Holm-adjusted significance are shown. *p < 0.05, **p < 0.01, ***p < 0.001. The corpus callosum and corticospinal ROIs are tract-based ROIs

*MCR-DIMWI* multi-compartment relaxometry-diffusion informed myelin water imaging, *METRICS* multi-echo T2 relaxation imaging with compressed sensing, *DTI* diffusion tensor imaging, *NODDI* neurite orientation dispersion and density imaging, *MWF* myelin water fraction, *IET2* geometrical mean of the intra- and extra-axonal T2, *MD* mean diffusivity, *RD* radial diffusivity, *AD* axial diffusivity, *FA* fractional anisotropy, *NDI* neurite density index, *ODI* orientation dispersion index, *FISO* free water fraction, *WM* white matter, *VWM* vanishing white matter, *MLD* metachromatic leukodystrophy

A group effect was also seen for NDI (*p* < 0.001, Table 2), with lower values in VWM and MLD than in controls (each *p* < 0.001), but no differences between VWM and MLD (*p* = 0.59). Only in the cerebellar white matter, NDI was similar to controls in VWM, whereas in MLD NDI was lower than in controls (Table 3 and Fig. 3).

Multi-level testing across ROIs showed a small group effect for FISO (*p* = 0.01, Table 2). FISO was highest in VWM (*p* = 0.04), and not significantly altered in MLD compared to controls (*p* = 0.99). Post-hoc testing showed that the difference in FISO between VWM and controls was driven by significant differences in frontal, parietal, and cerebellar white matter, the bundles through the corpus callosum, and corticospinal tract (Table 3 and Fig. 3).

ODI also differed by group (*p* < 0.001, Table 2). Overall, ODI was higher in VWM than in MLD (*p* = 0.002) and controls (*p* < 0.001), and higher in MLD than in controls (*p* = 0.02). In VWM, ODI was higher than in controls in all ROIs, while in MLD, ODI was similar to controls in frontal and occipital white matter and corticospinal tracts (Table 3 and Fig. S3).

Overall group effects were also seen for relaxation times T1, T2*, and IET2 (each *p* < 0.001, Table 2). The values were higher in VWM than in MLD and controls (each *p* < 0.001) and higher in MLD than in controls for T1 (*p* < 0.001), T2* (*p* < 0.01), and IET2 (*p* < 0.001).

DTI measures differed on a group level (each *p* < 0.001, Table 2). Mean diffusivity (MD) and radial diffusivity (RD) were both higher in VWM than in MLD (MD *p* = 0.03; RD *p* = 0.02) and controls (MD *p* < 0.001; RD *p* < 0.001), and higher in MLD than in controls (MD *p* < 0.001; RD *p* < 0.001). Axial diffusivity (AD) was higher in VWM (*p* < 0.001) and MLD (*p* = 0.004) than in controls, although temporal and cerebellar white matter did not show significant differences between groups (Table 3 and Fig. S3). AD did not differ between VWM and MLD (*p* = 0.13). FA was lower in VWM than in MLD and controls, and lower in MLD than in controls (all *p* < 0.001, Table 2). The DTI measures in temporal, occipital, and cerebellar white matter were often not significantly different between MLD and VWM (Table 3 and Fig. S3).

In VWM, the cerebellar white matter was mostly less affected than the cerebral white matter, as indicated by the smaller *t*-ratios of most quantitative measures indicating the age-corrected difference with controls (Table 3).

### METRICS T2-distribution

**Fig. 4:**
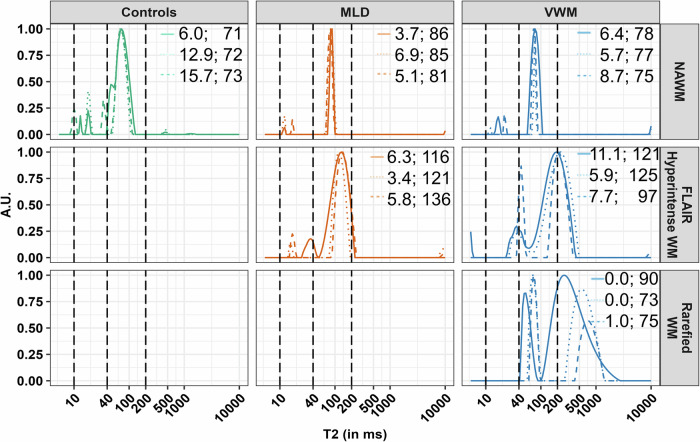
METRICS T2-distribution curves. T2-distribution curves in METRICS of three control subjects, three MLD and three VWM patients, from manually delineated ROIs in normal-appearing white matter, FLAIR hyperintense white matter (in VWM and MLD patients) and rarefied white matter (in VWM patients). The vertical lines represent the fitting boundaries of myelin water (10–40 ms) and intra- and extra-axonal water (40–200 ms). The MWF (in %) and IET2 (in ms) values of these ROIs are shown in the top-right corner of each panel. VWM, vanishing white matter; MLD, metachromatic leukodystrophy; MWF, myelin-water fraction; ROI, region-of-interest; METRICS, multi-echo T2 relaxation imaging with compressed sensing

Figure 4 shows examples of the METRICS T2-distribution curves of ROIs with normal-appearing, FLAIR-hyperintense, and FLAIR-hypointense (rarefied or cystic) white matter. These graphs show that with pathology, the T2-relaxation distribution is broadened and shifts to higher values in comparison to normal-appearing white matter. As a result, the myelin water peak and the intra- and extra-axonal water peak are crossing the preset boundaries of 40 ms and 200 ms.

### Clinical correlations

**Fig. 5:**
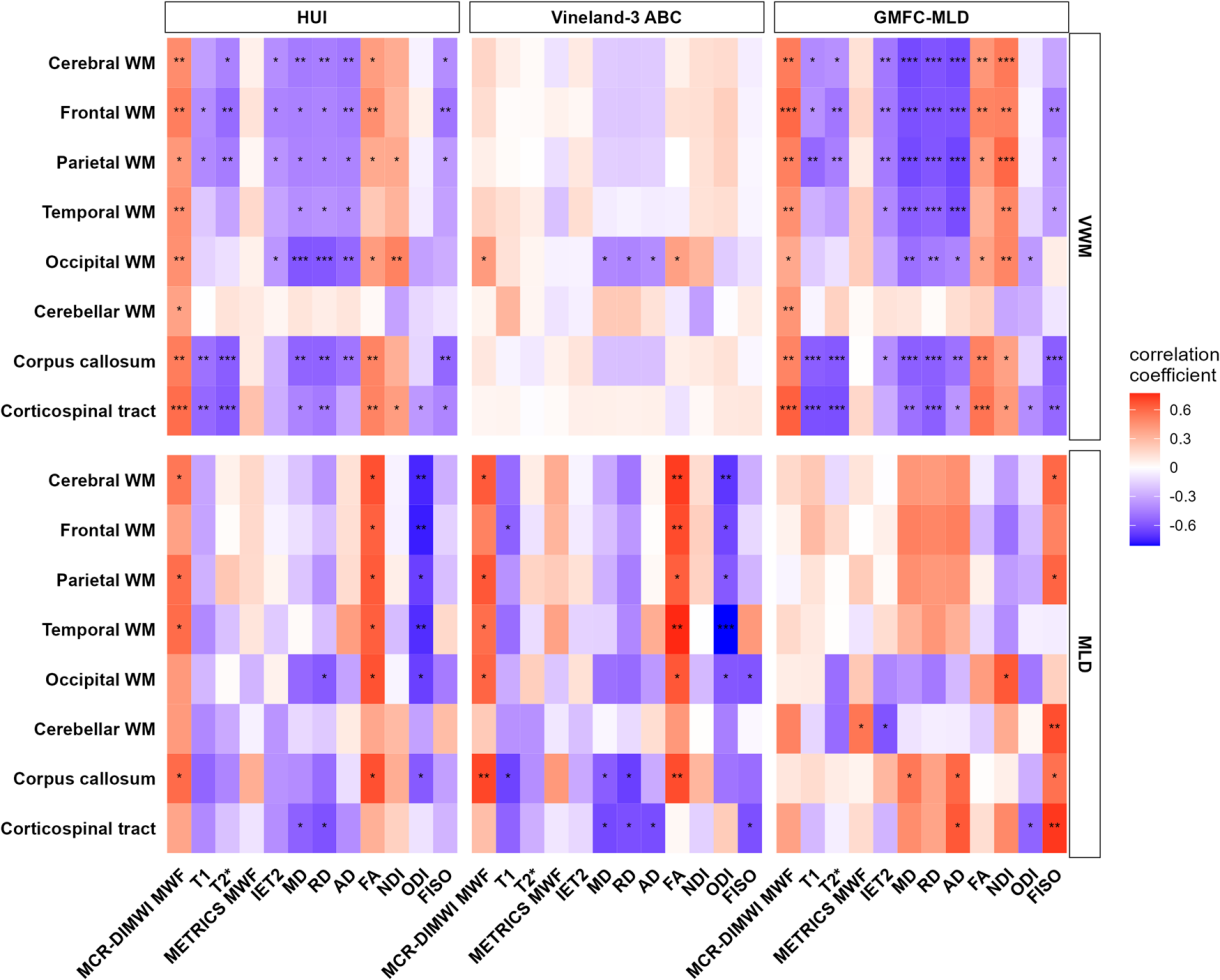
Correlations between MRI and clinical measures. Heat maps showing the correlation coefficients between MRI measures and clinical measures in VWM (**upper panels**) and MLD (**lower panels**). GMFC-MLD scores are inverted, to maintain similar directionality to HUI and Vineland scores (higher clinical scores indicate less affected patients). The corpus callosum and corticospinal ROIs are tract-based ROIs. *p < 0.05, **p < 0.01, ***p < 0.001. VWM, vanishing white matter; MLD, metachromatic leukodystrophy; ROI, region-of-interest; HUI, health utilities index

**Fig. 6:**
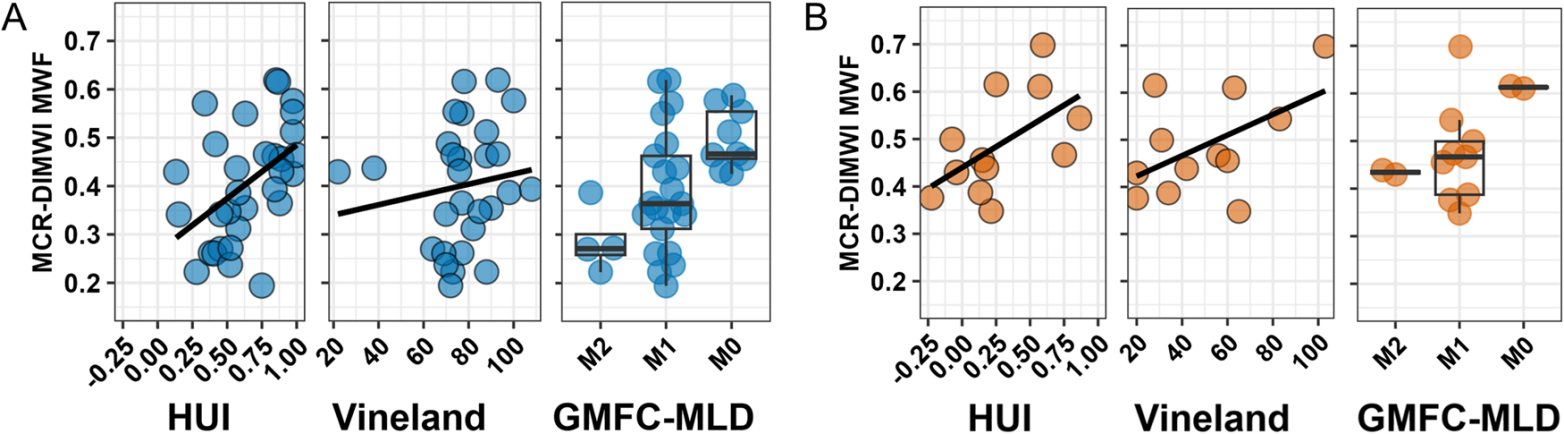
Scatterplots and boxplots between MWF ratio and clinical measures. MCR-DIMWI derived MWF-ratio in the entire cerebral white matter vs the Vineland, HUI, and GMFCS-MLD score in VWM (**A**) and MLD (**B**). VWM, vanishing white matter; MLD, metachromatic leukodystrophy; MWF, myelin-water fraction; MCR-DIMWI, multi-compartment relaxometry diffusion-informed myelin water imaging; HUI, health utilities index

Correlations between quantitative MRI measures and clinical measures are illustrated in Fig. 5. From the model-based quantitative MRI measures, MCR-DIMWI MWF correlated strongest with clinical measures in VWM and MLD (Fig. 5). Higher MCR-DIMWI MWF O/E ratios (i.e., MWF closer to normal) were related to better clinical function (higher HUI and Vineland and lower GMFC-MLD scores) in VWM (Fig. 6A) and MLD (Fig. 6B). NODDI-derived NDI and FISO also correlated with clinical measures, especially in VWM. The correlation coefficients between FISO and GMFC-MLD scores were opposite for VWM and MLD.

In VWM, DTI measures MD, RD, AD, and FA, and relaxation times T1, T2*, and IET2 also frequently correlated significantly to HUI scores and GMFC-MLD; correlation coefficients for Vineland-3 ABC were only occasionally significant. In MLD, correlation coefficients were often not statistically significant, but generally similar in magnitude and sign as in VWM, apart from the sign of several correlation coefficients between DTI measures, NDI, and FISO on the one hand and GMFC-MLD on the other hand.

## Discussion

This study confirms that quantitative brain white matter MRI measures can differentiate between controls and patients with VWM and MLD, and between VWM and MLD. For most measures, the direction of change relative to controls is similar for MLD and VWM, but the magnitude varies. Changes in relaxation times, MD, RD, FA, and ODI are more pronounced in VWM than in MLD. FISO is increased in VWM, but similar to controls in MLD. MWF, AD, and NDI differ between MLD and VWM in most white matter ROIs. These differential changes show that the diseases and related MRI changes are not part of a spectrum, but essentially different.

The quantitative MRI measures reflect known histopathological differences between VWM and MLD. White matter pathology in VWM has a rarifying nature [11], whereas in MLD accumulation of sulfatides and dense astrogliosis compensate for tissue loss, preserving tissue density [9]. These differences in pathology are reflected in the more pronounced elevation of white matter FISO and T1- and T2*- relaxation times in VWM than in MLD. The loss of all tissue components in VWM vs preservation of axons in MLD explains the significantly lower FA in VWM than in MLD. Loss of myelin and neurites are reflected by decreased MWF and NDI in both leukodystrophies. NDI is similar in VWM and MLD despite a higher water content in VWM. Based on diffusion characteristics, the free water compartment (FISO) is first determined by the NODDI model. NDI is the neurite fraction of the remaining tissue compartment [18]. Thus, high FISO values do not influence NDI directly. In literature, a tissue-weighted NDI is proposed as an alternative analysis, and would likely show larger differences between VWM and MLD [24]. In VWM cerebellar white matter, which is consistently less severely affected than cerebral white matter [12, 25, 26], several quantitative values are much closer to control values than in other regions or are within the normal range. In MLD, regional differences are less pronounced.

In our study, clinical scores are poorer in MLD than in VWM patients, whereas the opposite is true for quantitative MRI measures. In VWM, several quantitative measures correlate with clinical parameters. MCR-DIMWI MWF, NDI, and FA correlate positively; relaxation times, diffusivity measures, and FISO correlate negatively with GMFC-MLD and HUI scores for almost all ROIs. Although correlation coefficients with Vineland 3-ABC scores are not significant for most ROIs, the directionality of the relationships is mostly similar to that of HUI and GMFC-MLD scores. The smaller MLD group has less statistical power, but similar magnitude of correlation coefficients, suggesting that with more MLD patients more correlations would be significant. Interestingly, for several combinations of MRI and clinical measures, the direction of the correlation is opposite for VWM vs MLD. While high FISO is correlated to worse GMFC-MLD scores in VWM, the opposite is true in MLD. Thus, long-standing white matter pathology in MLD leads to lower FISO, indicating low free water content, while MWF tends to be lower, indicating loss of myelin. MLD causes sulfatide accumulation and dense astrogliosis [2]. The T2-pseudonormalization in advanced MLD is ascribed to lipid storage and indigestible myelin debris in macrophages [27]. While in our MLD patients, FISO is similar to controls, FISO in the study on T2-pseudonormalization was even lower than in controls, while NDI and MWF were also lower [27]. In line with this, in our MLD group, lower AD is correlated with worse GMFC-MLD scores, whereas the opposite is the case in VWM. AD in MLD is not only influenced by loss of myelin and axons (increasing AD), but also by intracellular storage of sulfatides (decreasing AD) [28]. Leukodystrophies are all characterized by individual pathological changes [2]. Our findings in VWM and MLD suggest that quantitative MRI measures will be differentially affected in other leukodystrophies, requiring evaluation of a combination of different quantitative MRI measures in each leukodystrophy before application.

We used model-free MRI measures, such as relaxation times and DTI measures, and model-based measures such as MWF, NDI, and FISO. Model-based measures are more readily translatable to microstructural components, but it is crucial to consider the possible effects of pathology on the estimated measures. Regarding MWF, the boundaries of what is considered myelin water are based on known relaxation characteristics of myelin water in healthy adult white matter or deducted from ex vivo studies [29, 30]. With pathology, myelin sheaths become less compact [31], which influences the relaxation times of myelin water. METRICS allows visualization of T2 distributions. In FLAIR hyperintense areas, the first peak, representing myelin water, extends beyond the 40 ms fitting boundary, the preset maximum T2 of myelin water. Consequently, part of this peak is not included in the MWF. In abnormal FLAIR hypointense areas, the first peak only starts after the 40 ms fitting boundary and MWF is 0%, although it is likely that there are still myelin sheaths present. It is possible to loosen the constraints, allowing the first peak to be fitted as MWF. However, this may lead to counter-intuitive results, as the MWF could even increase with pathology due to increased myelin water volume in less compact myelin. We therefore maintained the current fitting restrictions. Also, myelin debris may contribute to the myelin water signal [32]. This effect is especially important in leukodystrophies with insufficient clearance of myelin debris, like MLD.

Patient numbers are relatively large for ultra-rare diseases like VWM and MLD, but for analysis of covariates still larger numbers would be needed. The relatively low number of controls precluded individual matching for age at onset, disease duration, and sex. Sex differences in quantitative measures seem relatively unimportant, as they are much smaller [22] than the differences between controls and our patient cohorts. The impact of age, age at onset, and disease duration is complex. First, there are developmental changes in quantitative parameters, which we corrected by using deviation from the age-control line. Second, lower age at onset is associated with more severe disease and more rapid progression than later onset, meaning that neither age nor disease duration can straightforwardly be used as covariates. However, the quantitative MRI measures correlate with disease severity, thus inherently correcting the impact of age at onset and disease duration. Longitudinal data are needed to uncover the temporal evolution of quantitative MRI measures in individual patients and to determine correlations with clinical measures over time.

The imaging protocol used is quite lengthy. The omission of either MCR-DIMWI or METRICS would shorten the protocol by approximately 10 min. We chose to acquire both inherently different techniques, as comparable results would make our findings more robust. The sensitivity of both techniques for pathological changes is similar. The advantage of MCR-DIMWI is the stronger correlation with clinical measures, while the advantage of METRICS is that it facilitates visualization of the T2 distributions. More in-depth quantitative analyses on the peak location or full-width half maximum of myelin water could be performed in future studies.

Leukodystrophies share preferential white matter involvement, but each leukodystrophy is characterized by its individual underlying pathology details [2], which implies that quantitative measures could also be useful for diagnostic purposes. However, MRI pattern recognition using conventional sequences, combined with clinical information and genetic testing, is so powerful in rapidly establishing the correct diagnosis [3], that the contribution of quantitative measures will likely remain limited, also because these measures are dependent on disease stage and severity. There are still low numbers of leukodystrophies without a specific diagnosis and in those cases, quantitative MRI measures may be helpful in classifying them according to underlying pathology.

In conclusion, this study demonstrates that MRI measures quantitatively reflect different microstructural components of the underlying pathology and correlate with clinical measures. With that, they have the potential for a central role in leukodystrophy research. Different leukodystrophies have different types of underlying pathology and must be investigated separately, both regarding disease-specific changes in quantitative MRI measures and correlation with clinical findings [2]. While pattern recognition using conventional sequences will stay the cornerstone of diagnosing patients, quantitative MRI is a compelling candidate for monitoring patients, especially for providing outcome measures in the context of therapeutic trials.

## Supplementary information

ELECTRONIC SUPPLEMENTARY MATERIAL

## Abbreviations

AD: Axial diffusivity
FISO: Free (isotropic) water fraction
FLAIR: Fluid-attenuated inversion recovery
MCR-DIMWI: Multi-compartment relaxometry diffusion-informed myelin water imaging
MD: Mean diffusivity
METRICS: Multi-echo T2 relaxation imaging with compressed sensing
MLD: Metachromatic leukodystrophy
MWF: Myelin water fraction
NDI: Neurite density index
NODDI: Neurite orientation dispersion and density imaging
ODI: Orientation dispersion index
RD: Radial diffusivity
VWM: Vanishing white matter

## References

[1] Vanderver A, Prust M, Tonduti D et al (2015) Case definition and classification of leukodystrophies and leukoencephalopathies. Mol Genet Metab 114:494–500 25649058 10.1016/j.ymgme.2015.01.006 PMC4390457

[2] van der Knaap MS, Bugiani M (2017) Leukodystrophies: a proposed classification system based on pathological changes and pathogenetic mechanisms. Acta Neuropathol 134:351–382 28638987 10.1007/s00401-017-1739-1 PMC5563342

[3] van der Knaap MS, Schiffmann R, Mochel F, Wolf NI (2019) Diagnosis, prognosis, and treatment of leukodystrophies. Lancet Neurol 18:962–972 31307818 10.1016/S1474-4422(19)30143-7

[4] Stellingwerff MD, Pouwels PJW, Roosendaal SD, Barkhof F, van der Knaap MS (2023) Quantitative MRI in leukodystrophies. Neuroimage Clin 38:103427 37150021 10.1016/j.nicl.2023.103427 PMC10193020

[5] Stellingwerff MD, Al-Saady ML, Chan KS et al (2023) Applicability of multiple quantitative magnetic resonance methods in genetic brain white matter disorders. J Neuroimaging. 10.1111/jon.13167 10.1111/jon.13167 37925602

[6] Hamilton EMC, van der Lei HDW, Vermeulen G et al (2018) Natural history of vanishing white matter. Ann Neurol 84:274–288 30014503 10.1002/ana.25287 PMC6175238

[7] Schmidt JL, Pizzino A, Nicholl J et al (2020) Estimating the relative frequency of leukodystrophies and recommendations for carrier screening in the era of next‐generation sequencing. Am J Med Genet A 182:1906–1912 32573057 10.1002/ajmg.a.61641 PMC11348680

[8] Slynko I, Nguyen S, Hamilton EMC et al (2021) Vanishing white matter: eukaryotic initiation factor 2B model and the impact of missense mutations. Mol Genet Genomic Med. 10.1002/mgg3.1593:e1593 10.1002/mgg3.1593 PMC8104162 33432707

[9] Gieselmann V, Krageloh-Mann I (2010) Metachromatic leukodystrophy-an update. Neuropediatrics 41:1–6 20571983 10.1055/s-0030-1253412

[10] Abbink TEM, Wisse LE, Jaku E et al (2019) Vanishing white matter: deregulated integrated stress response as therapy target. Ann Clin Transl Neurol 6:1407–1422 31402619 10.1002/acn3.50826 PMC6689685

[11] Bugiani M, Vuong C, Breur M, van der Knaap MS (2018) Vanishing white matter: a leukodystrophy due to astrocytic dysfunction. Brain Pathol 28:408–421 29740943 10.1111/bpa.12606 PMC8028328

[12] Stellingwerff MD, Al-Saady ML, van de Brug T, Barkhof F, Pouwels PJW, van der Knaap MS (2021) MRI natural history of the leukodystrophy vanishing white matter. Radiology. 10.1148/radiol.2021210110:210110 10.1148/radiol.2021210110 34184934

[13] Furlong WJ, Feeny DH, Torrance GW, Barr RD (2001) The health utilities index (HUI®) system for assessing health-related quality of life in clinical studies. Ann Med 33:375–384 11491197 10.3109/07853890109002092

[14] Farmer C, Adedipe D, Bal V, Chlebowski C, Thurm A (2020) Concordance of the Vineland adaptive behavior scales, second and third editions. J Intellect Disabil Res 64:18–26 31657503 10.1111/jir.12691 PMC6941197

[15] Gavazzi F, Patel V, Charsar B et al (2023) Gross motor function in pediatric onset TUBB4A-related leukodystrophy: GMFM-88 performance and validation of GMFC-MLD in TUBB4A. J Child Neurol 38:498–504 37461315 10.1177/08830738231188159 PMC10527384

[16] Chan KS, Marques JP (2020) Multi-compartment relaxometry and diffusion informed myelin water imaging—promises and challenges of new gradient echo myelin water imaging methods. Neuroimage 221:117159 32663644 10.1016/j.neuroimage.2020.117159

[17] Dvorak AV, Wiggermann V, Gilbert G et al (2020) Multi-spin echo T2 relaxation imaging with compressed sensing (METRICS) for rapid myelin water imaging. Magn Reson Med 84:1264–1279 32065474 10.1002/mrm.28199

[18] Zhang H, Schneider T, Wheeler-Kingshott CA, Alexander DC (2012) NODDI: practical in vivo neurite orientation dispersion and density imaging of the human brain. Neuroimage 61:1000–1016 22484410 10.1016/j.neuroimage.2012.03.072

[19] Billot B, Greve DN, Puonti O et al (2023) SynthSeg: segmentation of brain MRI scans of any contrast and resolution without retraining. Med Image Anal 86:102789 36857946 10.1016/j.media.2023.102789 PMC10154424

[20] Zhang H, Yushkevich PA, Alexander DC, Gee JC (2006) Deformable registration of diffusion tensor MR images with explicit orientation optimization. Med Image Anal 10:764–785 16899392 10.1016/j.media.2006.06.004

[21] Garyfallidis E, Cote MA, Rheault F et al (2018) Recognition of white matter bundles using local and global streamline-based registration and clustering. Neuroimage 170:283–295 28712994 10.1016/j.neuroimage.2017.07.015

[22] Lebel C, Treit S, Beaulieu C (2019) A review of diffusion MRI of typical white matter development from early childhood to young adulthood. NMR Biomed 32:e3778 28886240 10.1002/nbm.3778

[23] Dean DC 3rd, O’Muircheartaigh J, Dirks H et al (2014) Modeling healthy male white matter and myelin development: 3 through 60months of age. Neuroimage 84:742–752 24095814 10.1016/j.neuroimage.2013.09.058 PMC3895775

[24] Parker CS, Veale T, Bocchetta M et al (2021) Not all voxels are created equal: reducing estimation bias in regional NODDI metrics using tissue-weighted means. Neuroimage 245:118749 34852276 10.1016/j.neuroimage.2021.118749 PMC8752961

[25] Stellingwerff MD, van de Wiel MA, van der Knaap MS (2022) Radiological correlates of episodes of acute decline in the leukodystrophy vanishing white matter. Neuroradiology. 10.1007/s00234-022-03097-3 10.1007/s00234-022-03097-3 36574026

[26] van der Lei HD, Steenweg ME, Barkhof F et al (2012) Characteristics of early MRI in children and adolescents with vanishing white matter. Neuropediatrics 43:22–26 22430157 10.1055/s-0032-1307456

[27] Martin P, Hagberg GE, Schultz T et al (2020) T2-pseudonormalization and microstructural characterization in advanced stages of late-infantile metachromatic leukodystrophy. Clin Neuroradiol 31:969–980 33226437 10.1007/s00062-020-00975-2 PMC8648649

[28] van Rappard DF, Konigs M, Steenweg ME et al (2018) Diffusion tensor imaging in metachromatic leukodystrophy. J Neurol 265:659–668 29383515 10.1007/s00415-018-8765-3 PMC5834549

[29] Odrobina EE, Lam TY, Pun T, Midha R, Stanisz GJ (2005) MR properties of excised neural tissue following experimentally induced demyelination. NMR Biomed 18:277–284 15948233 10.1002/nbm.951

[30] Dvorak AV, Swift-LaPointe T, Vavasour IM et al (2021) An atlas for human brain myelin content throughout the adult life span. Sci Rep 11:269 33431990 10.1038/s41598-020-79540-3 PMC7801525

[31] Klok MD, Bugiani M, de Vries SI et al (2018) Axonal abnormalities in vanishing white matter. Ann Clin Transl Neurol 5:429–444 29687020 10.1002/acn3.540 PMC5899913

[32] Webb S, Munro CA, Midha R, Stanisz GJ (2003) Is multicomponent T2 a good measure of myelin content in peripheral nerve? Magn Reson Med 49:638–645 12652534 10.1002/mrm.10411

