# Supplementary material for: Quantitative MRI distinguishes different leukodystrophies and correlates with clinical measures
Source: Eur Radiol. 2024 Sep 25;35(4):1845–57. doi: 10.1007/s00330-024-11089-5 (PMC11914348; doi:10.1007/s00330-024-11089-5)
Supplement: Supplementary file 1 — ELECTRONIC SUPPLEMENTARY MATERIAL [file 330_2024_11089_MOESM1_ESM.pdf]

Quantitative MRI distinguishes different leukodystrophies and correlates with clinical measures

Electronic Supplementary Material (ESM)

Eur Radiol (2024) Stellingwerff MD, Al-Saady ML, Chan K-S et al.

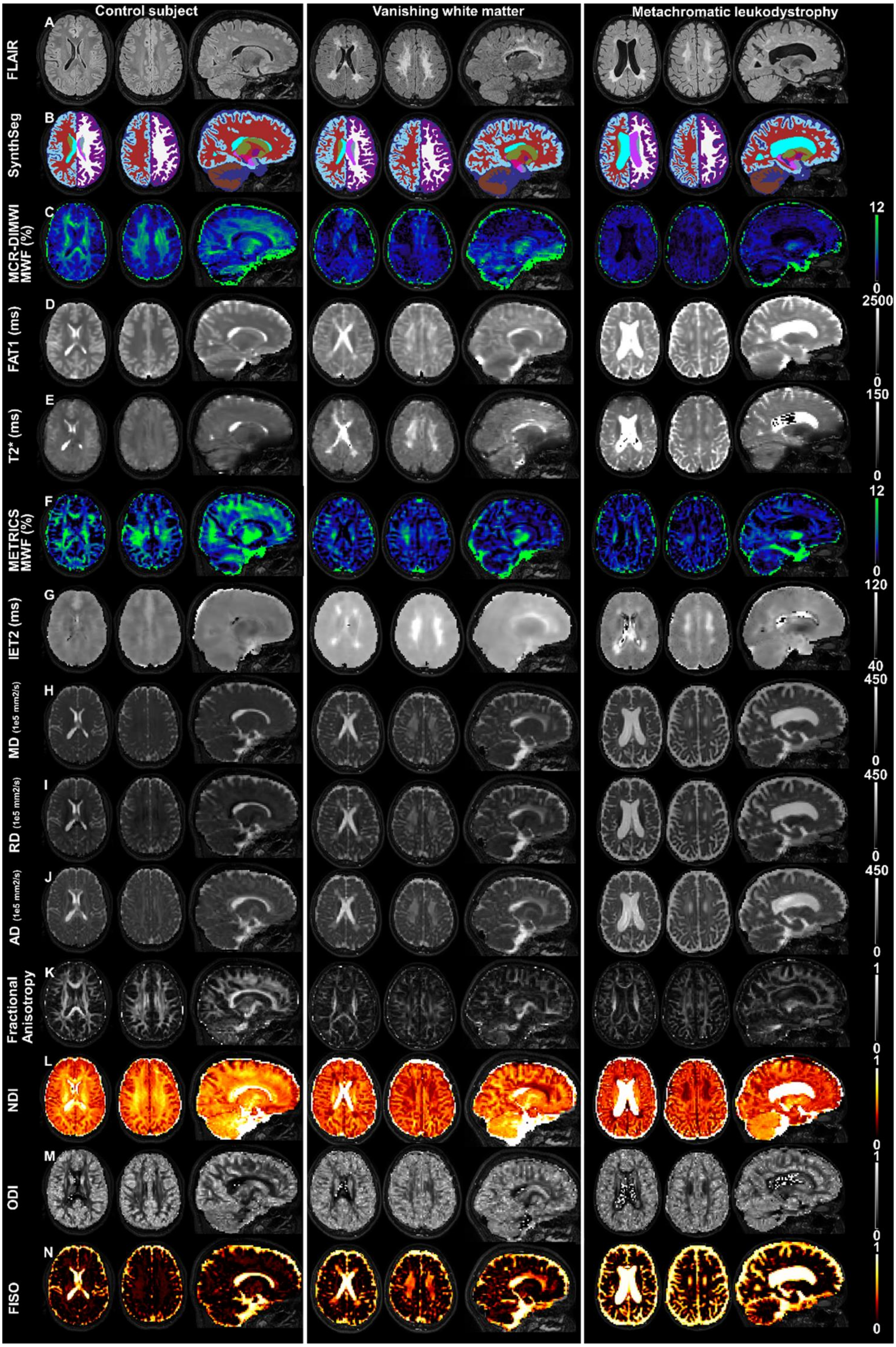

Fig.S1: Maps of quantitative parameters

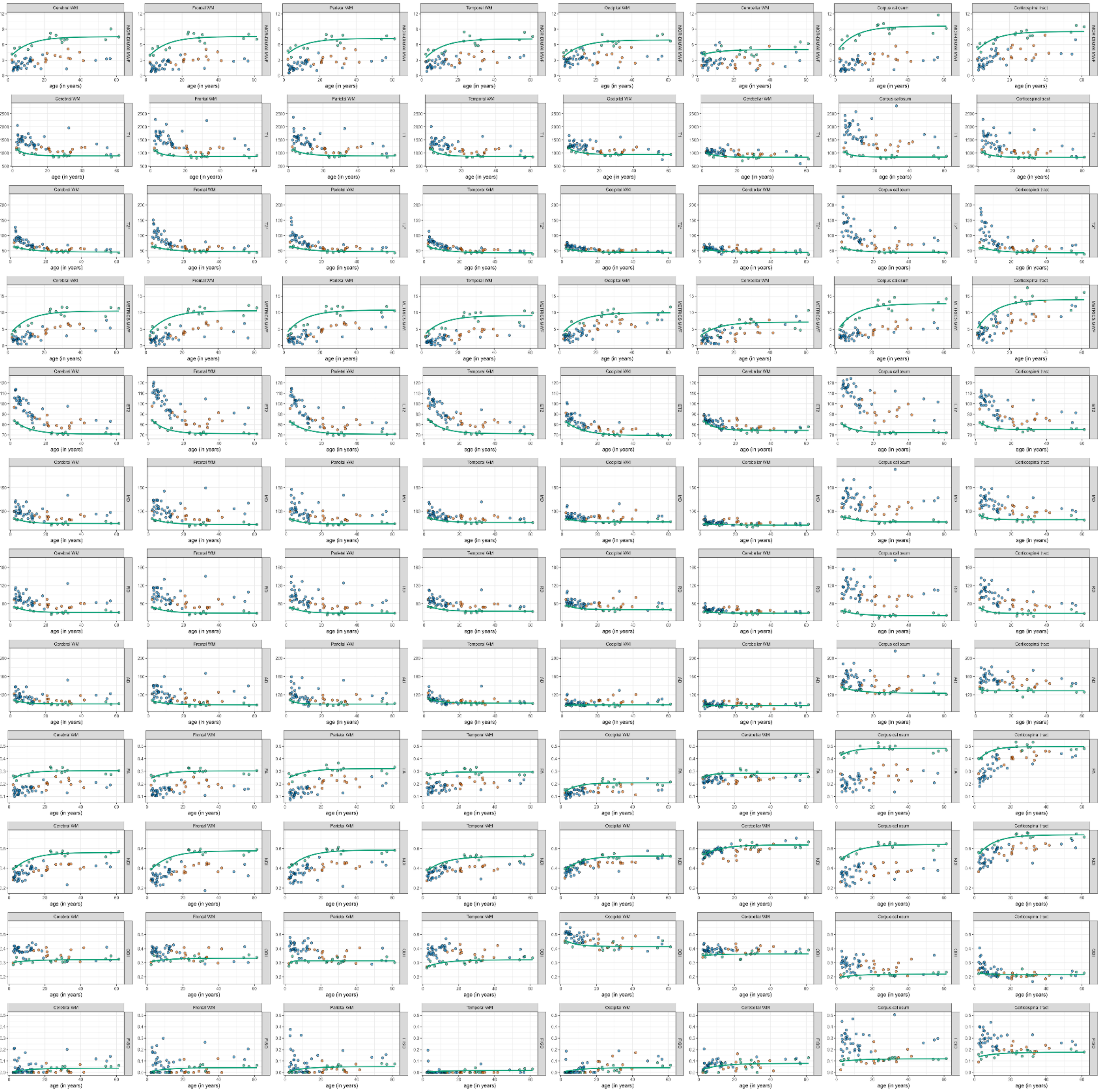

Fig.S2: Non-corrected data versus age and mono-exponential fits.

The corpus callosum and corticospinal ROIs are tract-based ROIs.

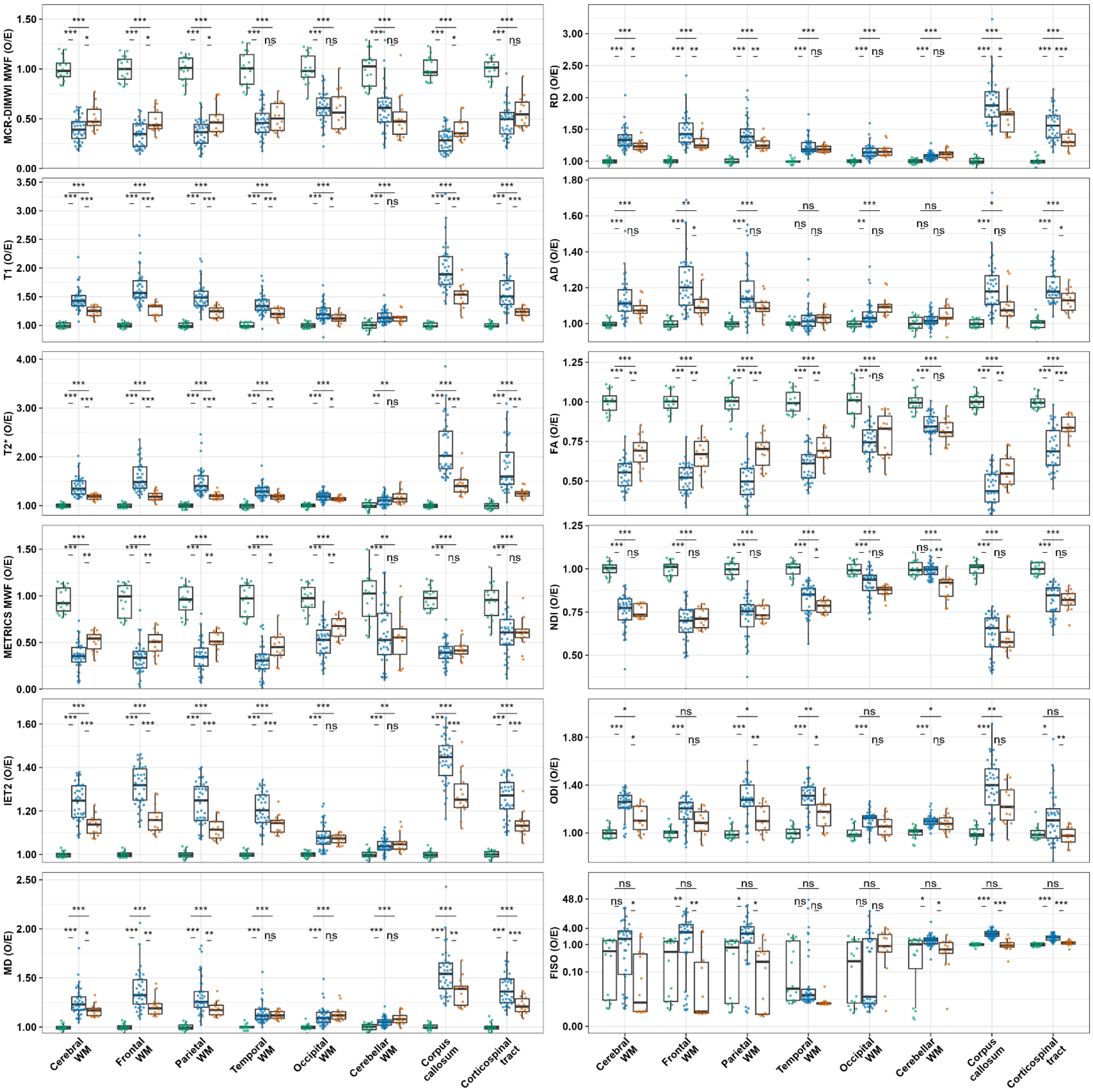

Fig.S3. Additional boxplots of age-corrected quantitative MRI data

Boxplots of the ratio of the observed and expected value (O/E) in controls (green), patients with VWM (blue) and patients with MLD (orange). ns = not significant, * = p<0.05, ** = p<0.01, *** = p<0.001. Of note: The scale for FISO is logarithmic. The corpus callosum and corticospinal ROIs are tract-based ROIs.

Table S1. Quantitative MRI measures in controls, MLD and VWM.

	group	MCR-DIMWI			METRICS		DTI	NODDI		
		MWF (%)	T1 (ms)	T2* (ms)	MWF (%)	IET2 (ms)	FA	NDI	ODI	FISO
Cerebral WM	Control	6.51 ± 1.45	946 ± 94	51 ± 7	8.49 ± 2.89	74 ± 5	0.29 ± 0.03	0.51 ± 0.06	0.32 ± 0.02	0.03 ± 0.02
	VWM	2.24 ± 0.83	1454 ± 219	79 ± 18	2.59 ± 1.50	96 ± 10	0.15 ± 0.03	0.35 ± 0.06	0.39 ± 0.03	0.06 ± 0.06
	MLD	3.46 ± 0.78	1136 ± 73	58 ± 6	4.87 ± 1.42	83 ± 6	0.20 ± 0.03	0.40 ± 0.04	0.36 ± 0.04	0.01 ± 0.03
Frontal WM	Control	6.52 ± 1.50	918 ± 89	52 ± 7	8.52 ± 3.07	75 ± 5	0.29 ± 0.03	0.52 ± 0.07	0.33 ± 0.02	0.03 ± 0.03
	VWM	1.96 ± 0.86	1572 ± 282	91 ± 24	2.37 ± 1.37	102 ± 10	0.14 ± 0.03	0.32 ± 0.06	0.37 ± 0.04	0.09 ± 0.08
	MLD	3.27 ± 0.84	1134 ± 94	60 ± 7	4.74 ± 1.69	85 ± 6	0.20 ± 0.03	0.39 ± 0.05	0.36 ± 0.04	0.02 ± 0.04
Parietal WM	Control	6.35 ± 1.27	953 ± 98	52 ± 7	8.65 ± 3.10	74 ± 4	0.30 ± 0.04	0.53 ± 0.07	0.31 ± 0.02	0.03 ± 0.03
	VWM	2.02 ± 0.90	1524 ± 262	86 ± 24	2.66 ± 1.68	96 ± 11	0.14 ± 0.04	0.35 ± 0.07	0.40 ± 0.04	0.09 ± 0.10
	MLD	3.23 ± 0.93	1138 ± 92	60 ± 6	5.07 ± 1.56	81 ± 6	0.21 ± 0.03	0.41 ± 0.05	0.35 ± 0.04	0.02 ± 0.03
Temporal WM	Control	6.06 ± 1.60	936 ± 103	49 ± 8	7.36 ± 2.66	75 ± 5	0.29 ± 0.02	0.48 ± 0.06	0.31 ± 0.02	0.01 ± 0.01
	VWM	2.42 ± 0.93	1350 ± 203	72 ± 14	1.99 ± 1.41	95 ± 10	0.17 ± 0.03	0.37 ± 0.05	0.38 ± 0.04	0.01 ± 0.04
	MLD	3.28 ± 0.89	1090 ± 76	56 ± 7	3.63 ± 1.15	84 ± 6	0.21 ± 0.03	0.39 ± 0.04	0.36 ± 0.04	0.00 ± 0.00
Occipital WM	Control	5.97 ± 1.38	1002 ± 102	48 ± 5	8.18 ± 2.63	73 ± 5	0.19 ± 0.03	0.49 ± 0.05	0.43 ± 0.03	0.02 ± 0.03
	VWM	3.10 ± 0.85	1283 ± 170	61 ± 7	3.56 ± 1.78	83 ± 7	0.13 ± 0.03	0.41 ± 0.06	0.48 ± 0.04	0.04 ± 0.05
	MLD	3.63 ± 1.08	1089 ± 80	54 ± 5	6.06 ± 1.58	77 ± 5	0.16 ± 0.03	0.44 ± 0.04	0.44 ± 0.03	0.06 ± 0.05
Cerebellar WM	Control	4.76 ± 0.92	879 ± 91	48 ± 5	5.94 ± 2.21	77 ± 3	0.28 ± 0.02	0.61 ± 0.05	0.36 ± 0.02	0.06 ± 0.05
	VWM	2.81 ± 1.20	1041 ± 115	55 ± 7	3.04 ± 2.08	82 ± 4	0.23 ± 0.02	0.58 ± 0.05	0.39 ± 0.02	0.08 ± 0.04
	MLD	2.50 ± 1.17	975 ± 79	55 ± 7	3.74 ± 2.03	80 ± 4	0.23 ± 0.02	0.55 ± 0.06	0.39 ± 0.03	0.06 ± 0.04
Corpus callosum	Control	8.38 ± 1.75	889 ± 79	49 ± 6	10.48 ± 3.16	74 ± 3	0.47 ± 0.03	0.60 ± 0.06	0.21 ± 0.01	0.11 ± 0.02
	VWM	2.09 ± 0.97	1830 ± 374	116 ± 37	3.42 ± 1.28	109 ± 10	0.21 ± 0.05	0.35 ± 0.07	0.29 ± 0.04	0.27 ± 0.08
	MLD	3.47 ± 0.94	1307 ± 158	68 ± 12	4.90 ± 1.50	94 ± 8	0.27 ± 0.05	0.37 ± 0.06	0.27 ± 0.04	0.12 ± 0.06
Corticospinal tract	Control	7.52 ± 1.43	881 ± 80	49 ± 7	11.12 ± 4.36	77 ± 3	0.47 ± 0.04	0.69 ± 0.07	0.22 ± 0.01	0.16 ± 0.03
	VWM	3.25 ± 1.44	1463 ± 310	99 ± 36	5.46 ± 2.90	98 ± 9	0.32 ± 0.08	0.52 ± 0.08	0.25 ± 0.05	0.29 ± 0.06
	MLD	4.50 ± 1.38	1055 ± 62	59 ± 7	7.52 ± 2.63	86 ± 5	0.41 ± 0.05	0.58 ± 0.07	0.21 ± 0.02	0.20 ± 0.04

Data are displayed as mean ± standard deviation. The corpus callosum and corticospinal ROIs are tract-based ROIs.
MCR-DIMWI = multi-compartment relaxometry-diffusion informed myelin water imaging, METRICS = multi-echo T2 relaxation imaging with compressed sensing, DTI = diffusion tensor imaging, NODDI = neurite orientation dispersion and density imaging, MWF = myelin water fraction, ms = millisecond, IET2 = geometrical mean of the intra- and extra-axonal T2, FA = fractional anisotropy, NDI = neurite density index, ODI = orientation dispersion index, FISO = free water fraction, WM=white matter, VWM = vanishing white matter, MLD = metachromatic leukodystrophy

Table S2: Ratio between the observed and age-expected value (O/E) per MRI measure.

	group	MCR-DIMWI			METRICS		DTI	NODDI		
		MWF (O/E)	T1 (O/E)	T2* (O/E)	MWF (O/E)	IET2 (O/E)	FA (O/E)	NDI (O/E)	ODI (O/E)	FISO (O/E)
Cerebral WM	Control	1.00 ± 0.12	1.00 ± 0.05	1.00 ± 0.04	0.96 ± 0.14	1.00 ± 0.01	1.00 ± 0.07	1.00 ± 0.04	1.00 ± 0.05	0.72 ± 0.67
	VWM	0.40 ± 0.13	1.46 ± 0.21	1.40 ± 0.21	0.36 ± 0.15	1.24 ± 0.09	0.55 ± 0.09	0.75 ± 0.10	1.24 ± 0.11	3.35 ± 5.27
	MLD	0.51 ± 0.12	1.24 ± 0.09	1.18 ± 0.06	0.52 ± 0.11	1.13 ± 0.05	0.68 ± 0.10	0.75 ± 0.04	1.12 ± 0.11	0.42 ± 0.79
Frontal WM	Control	1.00 ± 0.12	1.00 ± 0.05	1.00 ± 0.05	0.95 ± 0.18	1.00 ± 0.01	1.00 ± 0.07	1.00 ± 0.04	1.00 ± 0.05	0.72 ± 0.72
	VWM	0.34 ± 0.13	1.64 ± 0.28	1.59 ± 0.31	0.33 ± 0.16	1.31 ± 0.09	0.52 ± 0.09	0.68 ± 0.12	1.17 ± 0.12	4.55 ± 5.29
	MLD	0.48 ± 0.11	1.27 ± 0.12	1.19 ± 0.10	0.50 ± 0.13	1.16 ± 0.06	0.66 ± 0.11	0.72 ± 0.06	1.08 ± 0.11	0.53 ± 1.09
Parietal WM	Control	1.00 ± 0.13	1.00 ± 0.05	1.00 ± 0.06	0.95 ± 0.16	1.00 ± 0.02	1.00 ± 0.09	1.00 ± 0.04	1.00 ± 0.06	0.72 ± 0.64
	VWM	0.35 ± 0.13	1.51 ± 0.23	1.50 ± 0.29	0.36 ± 0.16	1.24 ± 0.10	0.50 ± 0.12	0.72 ± 0.12	1.28 ± 0.15	4.45 ± 6.39
	MLD	0.49 ± 0.15	1.23 ± 0.10	1.20 ± 0.07	0.52 ± 0.11	1.12 ± 0.05	0.69 ± 0.10	0.74 ± 0.05	1.12 ± 0.11	0.47 ± 0.67
Temporal WM	Control	1.00 ± 0.17	1.00 ± 0.04	1.00 ± 0.06	0.96 ± 0.17	1.00 ± 0.02	1.00 ± 0.07	1.00 ± 0.04	1.00 ± 0.05	0.62 ± 0.87
	VWM	0.47 ± 0.15	1.37 ± 0.18	1.30 ± 0.14	0.31 ± 0.17	1.21 ± 0.08	0.61 ± 0.11	0.83 ± 0.09	1.30 ± 0.14	2.12 ± 8.02
	MLD	0.52 ± 0.14	1.21 ± 0.09	1.19 ± 0.07	0.46 ± 0.15	1.14 ± 0.04	0.70 ± 0.09	0.78 ± 0.04	1.16 ± 0.12	0.01 ± 0.01
Occipital WM	Control	1.00 ± 0.15	1.00 ± 0.05	1.00 ± 0.05	0.97 ± 0.15	1.00 ± 0.01	1.00 ± 0.10	1.00 ± 0.04	1.00 ± 0.06	0.60 ± 0.72
	VWM	0.61 ± 0.16	1.22 ± 0.17	1.19 ± 0.09	0.51 ± 0.18	1.08 ± 0.06	0.76 ± 0.12	0.91 ± 0.09	1.11 ± 0.07	1.56 ± 3.37
	MLD	0.59 ± 0.20	1.12 ± 0.09	1.14 ± 0.05	0.67 ± 0.12	1.07 ± 0.02	0.79 ± 0.14	0.87 ± 0.03	1.05 ± 0.08	1.47 ± 1.37
Cerebellar WM	Control	1.00 ± 0.17	1.00 ± 0.08	1.00 ± 0.08	0.98 ± 0.26	1.00 ± 0.02	1.00 ± 0.07	1.00 ± 0.04	1.00 ± 0.05	0.87 ± 0.65
	VWM	0.62 ± 0.26	1.14 ± 0.14	1.10 ± 0.12	0.58 ± 0.31	1.04 ± 0.03	0.86 ± 0.07	0.99 ± 0.04	1.10 ± 0.05	1.61 ± 1.03
	MLD	0.51 ± 0.23	1.14 ± 0.09	1.17 ± 0.13	0.56 ± 0.28	1.05 ± 0.04	0.82 ± 0.08	0.89 ± 0.07	1.08 ± 0.07	0.80 ± 0.60
Corpus callosum	Control	1.00 ± 0.11	1.00 ± 0.05	1.00 ± 0.05	0.98 ± 0.12	1.00 ± 0.02	1.00 ± 0.05	1.00 ± 0.04	1.00 ± 0.05	1.00 ± 0.12
	VWM	0.28 ± 0.12	1.99 ± 0.40	2.18 ± 0.57	0.39 ± 0.13	1.43 ± 0.10	0.46 ± 0.10	0.62 ± 0.12	1.39 ± 0.23	2.59 ± 0.81
	MLD	0.40 ± 0.11	1.51 ± 0.21	1.44 ± 0.24	0.43 ± 0.10	1.28 ± 0.11	0.57 ± 0.10	0.59 ± 0.07	1.24 ± 0.17	1.07 ± 0.52
Corticospinal	Control	1.00 ± 0.10	1.00 ± 0.05	1.00 ± 0.07	0.95 ± 0.20	1.00 ± 0.02	1.00 ± 0.04	1.00 ± 0.03	1.00 ± 0.06	1.00 ± 0.14
tract	VWM	0.48 ± 0.17	1.59 ± 0.30	1.82 ± 0.53	0.58 ± 0.23	1.26 ± 0.09	0.70 ± 0.13	0.82 ± 0.09	1.13 ± 0.21	1.87 ± 0.47
	MLD	0.57 ± 0.16	1.23 ± 0.08	1.26 ± 0.10	0.60 ± 0.16	1.14 ± 0.06	0.84 ± 0.07	0.82 ± 0.06	0.97 ± 0.07	1.16 ± 0.22

Data are displayed as mean ± standard deviation. The corpus callosum and corticospinal ROIs are tract-based ROIs.
MCR-DIMWI = multi-compartment relaxometry-diffusion informed myelin water imaging, METRICS = multi-echo T2 relaxation imaging with compressed sensing, DTI = diffusion tensor imaging, NODDI = neurite orientation dispersion and density imaging, MWF = myelin water fraction, O/E = ratio observed and age-expected measure, IET2 = geometrical mean of the intra- and extra-axonal T2, FA = fractional anisotropy, NDI = neurite density index, ODI = orientation dispersion index, FISO = free water fraction, WM=white matter, VWM = vanishing white matter, MLD = metachromatic leukodystrophy
